# The neural signature of psychomotor disturbance in depression

**DOI:** 10.1038/s41380-023-02327-1

**Published:** 2023-12-01

**Authors:** Florian Wüthrich, Stephanie Lefebvre, Vijay A. Mittal, Stewart A. Shankman, Nina Alexander, Katharina Brosch, Kira Flinkenflügel, Janik Goltermann, Dominik Grotegerd, Tim Hahn, Hamidreza Jamalabadi, Andreas Jansen, Elisabeth J. Leehr, Susanne Meinert, Igor Nenadić, Robert Nitsch, Frederike Stein, Benjamin Straube, Lea Teutenberg, Katharina Thiel, Florian Thomas-Odenthal, Paula Usemann, Alexandra Winter, Udo Dannlowski, Tilo Kircher, Sebastian Walther

**Affiliations:** 1 Translational Research Center, University Hospital of Psychiatry and Psychotherapy, University of Bern, Bern, Switzerland; 2 Graduate School of Health Science, University of Bern, Bern, Switzerland; 3 Department of Psychiatry and Behavioral Sciences, Northwestern University, Chicago, IL USA; 4 Department of Psychology, Northwestern University, Evanston, IL USA; 5 Northwestern University, Institute for Innovations in Developmental Sciences, Evanston/Chicago, IL USA; 6 Northwestern University, Institute for Policy Research, Evanston, IL USA; 7 Northwestern University, Medical Social Sciences, Chicago, IL USA; 8 Department of Psychiatry and Psychotherapy, University of Marburg, Marburg, Germany; 9 Center for Mind, Brain and Behavior (CMBB), University of Marburg, Marburg, Germany; 10 Institute for Translational Psychiatry, University of Münster, Münster, Germany; 11 Core-Facility Brain imaging, Faculty of Medicine, University of Marburg, Marburg, Germany; 12 Institute for Translational Neuroscience, University of Münster, Münster, Germany

**Keywords:** Depression, Biomarkers

## Abstract

Up to 70% of patients with major depressive disorder present with psychomotor disturbance (PmD), but at the present time understanding of its pathophysiology is limited. In this study, we capitalized on a large sample of patients to examine the neural correlates of PmD in depression. This study included 820 healthy participants and 699 patients with remitted (*n* = 402) or current (*n* = 297) depression. Patients were further categorized as having psychomotor retardation, agitation, or no PmD. We compared resting-state functional connectivity (ROI-to-ROI) between nodes of the cerebral motor network between the groups, including primary motor cortex, supplementary motor area, sensory cortex, superior parietal lobe, caudate, putamen, pallidum, thalamus, and cerebellum. Additionally, we examined network topology of the motor network using graph theory. Among the currently depressed 55% had PmD (15% agitation, 29% retardation, and 11% concurrent agitation and retardation), while 16% of the remitted patients had PmD (8% retardation and 8% agitation). When compared with controls, currently depressed patients with PmD showed higher thalamo-cortical and pallido-cortical connectivity, but no network topology alterations. Currently depressed patients with retardation only had higher thalamo-cortical connectivity, while those with agitation had predominant higher pallido-cortical connectivity. Currently depressed patients without PmD showed higher thalamo-cortical, pallido-cortical, and cortico-cortical connectivity, as well as altered network topology compared to healthy controls. Remitted patients with PmD showed no differences in single connections but altered network topology, while remitted patients without PmD did not differ from healthy controls in any measure. We found evidence for compensatory increased cortico-cortical resting-state functional connectivity that may prevent psychomotor disturbance in current depression, but may perturb network topology. Agitation and retardation show specific connectivity signatures. Motor network topology is slightly altered in remitted patients arguing for persistent changes in depression. These alterations in functional connectivity may be addressed with non-invasive brain stimulation.

## Introduction

Major depressive disorder (MDD) is one of the most common psychiatric disorders with a lifetime prevalence of up to 21% [1, 2]. MDD is among the leading causes of burden of disease [3] and treatments for it have mixed efficacy. About half of the patients with MDD do not respond to first-line medication and up to a third of the patients remain treatment-resistant even after multiple therapeutic attempts [4–6]. This poor treatment response may be due to the large heterogeneity of symptoms that can be present in MDD [7, 8].

One of the frequent symptom domains of MDD is psychomotor disturbance (PmD). It occurs in up to 70% of patients and is associated with higher depression severity and poorer treatment response to antidepressants [9–13]. PmD presents in two dimensions: psychomotor retardation (PmR) and psychomotor agitation (PmA). PmR is characterized by a reduction of movement and activity, e.g., postural slumping, lower volume of voice, reduced facial expressions, or slowed gait. PmA represents an increased amount or amplitude of movements, as well as a shift toward a more erratic nature of motion, e.g., restlessness. Despite their seemingly opposite nature, PmR and PmA can occur simultaneously [14]. Moreover, PmD tends to persist in remitted patients [15]. To date, the pathophysiology of PmD remains elusive, and there are no treatments available for this specific feature of MDD.

MDD has been linked to various structural and functional brain alterations. Structural alterations encompass a global decrease of gray matter volume, particularly in cerebellum, limbic network, and multiple frontal areas [16]; as well as a cortical thinning in frontal, temporal, and limbic regions [17]. In addition, patients with MDD present with widespread lower fractional anisotropy, including the corona radiata, corpus callosum (genu and body but not splenium), external capsule, anterior limb of internal capsule, sagittal stratum, fronto-occipital fasciculus, cingulate part of the cingulum bundle, and stria terminalis [18]. Furthermore, functional alterations have been reported in intrinsic resting-state networks such as the default mode network or the salience network [19–21].

However, little is currently known on neural correlates of PmD in depression, specifically. Lower physical activity is linked to altered white matter integrity in motor circuits in MDD [22, 23], and to altered perfusion in orbitofrontal, rostral frontal cortex, and supplementary motor area (SMA) where higher cerebral blood flow is associated with reduced physical activity [24], while patients with MDD and PmR reportedly present lower cerebral blood flow in the primary motor cortex than patients without PmR [25].

Alterations of BOLD resting-state functional connectivity (rsFC) associated with PmD in MDD have not been explored. Three motor circuits that might be affected by motor disturbance have been proposed: (1), the cortico-basal ganglia circuit including primary motor cortex, caudate, putamen, pallidum, and thalamus; (2), the cerebello-thalamo-motor circuit, including primary motor cortex, thalamus, and cerebellum; and (3), the cortico-cortical motor circuit, including premotor and motor cortex, medial prefrontal cortex as well as parietal cortex [13, 26].

In the current study, we capitalized on a large cohort of healthy controls and patients with current or remitted MDD to explore the functional correlates of PmD at three levels: (i) diagnosis (MDD vs. controls), (ii) type of PmD in MDD (i.e., PmR or PmA), and (iii) PmD in distinct disorder states (current depression or remission). We examined resting-state functional connectivity and hypothesized PmD to be associated with altered connectivity within the 3 aforementioned motor circuits, especially in the cortical-subcortical connections. We also expected functional connectivity alterations associated with PmD to depend on depression states.

## Methods

### Participants

We included 1683 participants from the Marburg-Münster Affective Disorders Cohort Study (MACS) for which both structural and resting-state data were available. The MACS is part of the FOR2107 research consortium that studies risk factors, course of illness, and neurobiological correlates of major psychoses, including MDD [27]. The sample in the present study consisted of 769 participants with current or history of MDD and 914 healthy controls (HC). General descriptions of the cohort and MRI protocols have been published elsewhere [27, 28]. Clinical assessments were conducted by trained psychologists. Instruments included the Hamilton Depression Rating Scale (HAMD) [29], Beck’s Depression Inventory (BDI-I) [30], and the Global Assessment of Functioning (GAF) [31], all of which had excellent intraclass correlations [27].

Briefly, participants aged 18−65 years were recruited at Marburg and Münster Universities in Germany through advertisements and in psychiatric hospitals. Diagnosis of MDD according to DSM-IV or lack thereof was confirmed by trained psychologists using the SCID [32]. Exclusion criteria included history of serious neurological or medical disorders, substance abuse, contraindication for MRI, and current benzodiazepine/opioid/Z-drugs use. Clinical assessments and MR scans took place after the participants provided written informed consent. The study and all procedures have been approved by the respective local Ethics committees and are in accordance with the Declaration of Helsinki.

Participants with a lifetime diagnosis of MDD were categorized as currently depressed or remitted. Current depression was defined as HAMD score >8. We further divided patients into those with PmD (slowed (HAMD item 8 “retardation” ≥1), agitated (HAMD item 9 “agitation” ≥1), concurrent agitation and retardation (PmM, HAMD items 8 and 9 ≥ 1)), and those without PmD (HAMD item 8 and 9 = 0) groups. Trained experts evaluated psychomotor behavior of the participants during the interviews. The HAMD items 8 and 9 are not self-reported and thus free from recall bias.

We excluded 44 HC who scored at least 1 point in the PmD items of the Hamilton depression rating scale (HAMD), and 19 participants due to missing or incomplete HAMD. We additionally excluded 101 participants with a mean framewise displacement (mFD) > 0.5 mm in the resting-state sequence, resulting in a final sample of 1519 participants composed of 820 healthy participants and 699 patients (297 currently depressed, 402 remitted). Sample characteristics are reported in Table 1.

**Table 1:** Sample demographics.

	MDD currently depressed *N* = 297	MDD remitted *N* = 402	Healthy participants *N* = 820	Statistic	Post-hoc
Age (years)	35.2 ± 13.2	35.5 ± 13.0	33.5 ± 12.6	*F*_(2,1516)_ = 4.2 *p* = 0.015	cur-HC: *p* = 0.13 cur-rem: *p* = 0.93 rem-HC: *p* = 0.022
Sex N f (% f)	197 (66.4%)	265 (65.9%)	528 (64.4%)	Χ^2^ = .507 *p* = 0.78	
Education (years)	13.0 ± 2.7	13.6 ± 2.6	14.0 ± 2.6	*F*_(2,1490)_ = 17.3 *p* < 0.001	cur-HC: *p* < 0.001 cur-rem: *p* = 0.013 rem-HC: *p* = 0.010
HAMD17	14.6 ± 4.4	3.9 ± 3.4	1.3 ± 2.0	*F*_(2,1516)_ = 2164 *p* < 0.001	cur-HC: *p* < 0.001 cur-rem: *p* < 0.001 rem-HC: *p* < 0.001
BDI-I	24.6 ± 9.5	11.7 ± 8.7	4.0 ± 4.1	*F*_(2,1482)_ = 967.8 *p* < 0.001	cur-HC: *p* < 0.001 cur-rem: *p* < 0.001 rem-HC: *p* < .001
GAF	56.9 ± 9.8	71.9 ± 12.7	91.6 ± 7.0	*F*_(2,1496)_ = 1628 *p* < 0.001	cur-HC: *p* < 0.001 cur-rem: *p* < 0.001 rem-HC: *p* < 0.001

*Cur* currently depressed patients, *rem* remitted patients, *HC* healthy controls, *MDD* major depressive disorder, *HAMD17* Hamilton depression rating scale, 17 item version, *BDI-I* revised Beck depression inventory, *GAF* Global assessment of functioning.

### Data acquisition

MRI scans were acquired at both centers using 3 T Siemens scanners (Marburg: Tim Trio, Siemens, Erlangen, Germany, 12-channel head coil; Münster: Prisma, Siemens, Erlangen, Germany, 20-channel head coil). Sequences were kept as similar as possible across the two centers. One hardware change took place in Marburg during the study (replacement of a defective gradient coil) and one software change in Münster (switch on of pre-scan normalization) [28]. At both centers, a high resolution T1 scan (MPRAGE, voxelsize = 1 x 1x 1 mm^3^, TI = 900 ms, Marburg: TR = 1900 ms, TE = 2.26 ms, FA = 9°, 176 sagittal slices; Münster: TR = 2130 ms, TE = 2.28 ms, FA = 9°, 192 sagittal slices) and 8 min of resting-state fMRI (T2* EPI, 237 volumes, voxelsize =3.28 × 3.28 × 4.18 mm, TR = 2000 ms, FA = 90°, Marburg: TE = 30 ms; Münster: TE = 29 ms) were acquired. Results of functional connectivity analyses in this cohort have been recently reported in relation to different aims [33, 34].

### Image preprocessing

Resting-state data preprocessing and analyses were carried out in the CONN toolbox 20.b (www.nitrc.org/projects/conn) under Matlab 2020b (MathWorks, Natick, USA). We followed the standard preprocessing pipeline in the CONN toolbox including realignment, slice-timing correction, direct segmentation and normalization, and smoothing with a 5 mm FWHM kernel. Subjects with mFD > 0.5 mm were excluded. Denoising included regressing out noise from white matter and cerebrospinal fluid signals, motion regression, temporal bandpass filtering (0.008−0.09 Hz), and detrending, similar to the preprocessing pipelines in previous studies [33, 34]. We performed the analyses with and without scrubbing (thresholds: global-signal z-value: 5, motion: 0.9 mm), as well as with and without HC presenting some level of PmD. Since results did not differ substantially, we only report the analyses without scrubbing and excluding HC subjects with PmD.

### Analyses

Statistical analysis of behavioral data was carried out in R 4.0.3 (R Core Team, https://www.R-project.org/). We compared age, sex distribution, education, depression severity (i.e., BDI-I and HAMD-sums), and global functioning between HC, currently depressed, and remitted patients (Table 1), and between PmD groups in currently depressed and remitted patients separately (Table 2) using ANOVAs and Chi-square tests, where appropriate. To compare the HAMD-sums, we subtracted the two PmD items from the sum, as we used these items for categorization and, thus biased the PmD groups toward higher sum-scores.

**Table 2:** Characterization of PmD groups by depressive state.

Currently Depressed MDD						
	PmR *N* = 87 (29.3%)	PmM *N* = 34 (11.4%)	PmA *N* = 44 (14.8%)	nonPmD *N* = 132 (44.4%)	Statistic	Post-hoc
Age years	34.3 ± 13.0	37.0 ± 15.1	34.1 ± 14.5	36.2 ± 12.9	*F*_(3293)_ = 0.66 *p* = .58	
Sex % female	54 (66.7%)	17 (55.9%)	25 (61.4%)	94 (72.0%)	Χ^2^ = 4.01 *p* = 0.26	
Education years	13.1 ± 2.6	11.9 ± 2.4	13.1 ± 3.1	13.2 ± 2.7	*F*_(3287)_ = 2.07 *p* = 0.11	
HAMD-PmD	13.8 ± 4.3	14.5 ± 5.1	13.9 ± 4.7	13.3 ± 3.4	*F*_(3293)_ = 0.80 *p* = 0.50	
BDI-I	24.7 ± 9.9	26.3 ± 8.6	25.7 ± 11.7	23.7 ± 8.7	*F*_(3282)_ = 0.94 *p* = 0.42	
GAF	55.9 ± 9.9	54.4 ± 7.8	55.9 ± 10.1	58.6 ± 10.3	*F*_(3287)_ = 2.42 *p* = 0.067	nonPmD vs: PmR: *p* = 0.053 PmM: *p* = 0.013 PmA: *p* = 0.14

Remitted MDD						
	PmR *N* = 32 (8.0%)	PmM *N* = 2 (0.5%)	PmA *N* = 32 (8.0%)	nonPmD *N* = 336 (83.6%)	Statistic	Post-hoc
Age	35.2 ± 12.6		35.6 ± 12.2	35.5 ± 12.9	*F*_(2397)_ = 0.009 *p* = 0.99	
Sex	17 (53.1%)		15 (46.9%)	230 (68.5%)	Χ^2^ = 6.59 *P* = 0.037	nonPmD vs: PmR: *p* = 0.080 PmA: *p* = 0.041
Education	13.3 ± 2.8		12.6 ± 2.7	13.7 ± 2.6	*F*_(2286)_ = 2.68 *p* = 0.07	nonPmD vs: PmR *p* = 0.37 PmA: *p* = 0.038 PmR vs PmA: *p* = 0.38
HAMD-PmD	4.0 ± 2.4		3.5 ± 2.1	3.3 ± 2.7	*F*_(2397)_ = 1.16 *p* = 0.31	
BDI-I	14.6 ± 10.0		12.0 ± 8.4	11.4 ± 8.6	*F*_(2390)_ = 1.93 *p* = 0.15	
GAF	63.5 ± 12.7		68.2 ± 9.9	73.5 ± 12.2	*F*_(2393)_ = 12.0 *p* < 0.001	nonPmD vs: PmR: *p* < 0.001 PmA: *p* = 0.007 PmA v PmR : *p* = 0.11

Comparison of demographics and clinical characteristics between the different psychomotor disturbance groups in currently depressed and remitted patients separately. No comparisons with remitted patients with psychomotor retardation and agitation due to group size (*N* = 1).

*MDD* major depressive disorder, *PmD* psychomotor disturbance, *PmR* psychomotor retardation, *PmA* psychomotor agitation, *PmM* concurrent psychomotor agitation and retardation, *HAMD-PmD* Hamilton depression rating scale without the retardation and agitation items, *BDI-I* revised Beck depression inventory, *GAF* global assessment of functioning.

To examine whether resting-state BOLD connectivity within the cerebral motor network is altered by MDD and PmD, we performed a ROI-to-ROI analysis with 18 anatomical ROIs covering cortical and subcortical regions of the motor network: Left and right primary motor (M1) and sensory (S1) cortices, superior parietal lobule, thalamus, caudate, putamen, pallidum, SMA from the Harvard-Oxford atlases [35–38], and cerebellar lobules 4 and 5 (the lobules classically most associated with motor function, as one ROI) from the AAL atlas [39]. We extracted timeseries for each ROI and cross-correlated them for each participant using bivariate correlations. Then we compared the resulting z-transformed correlation matrices between groups as specified below. Connection-level hypotheses were evaluated using multivariate parametric statistics with random-effects across subjects and sample covariance estimation across multiple measurements. Inferences were performed at the level of individual clusters (groups of similar connections). Cluster-level inferences considered groups of related ROIs identified using a complete-linkage hierarchical clustering procedure [40] based on ROI-to-ROI anatomical proximity and functional similarity metrics [41]. Once these groups of ROIs were defined, the entire set of connections between all pairs of ROIs *within*- and *between*- group connectivity sets was analyzed.

To examine whether the efficiency of this predefined cerebral motor network is altered by MDD and PmD, we performed a graph theory analysis (as implemented in the CONN toolbox) using a cost-thresholded network with the above-mentioned ROIs as nodes and their two-sided correlations as edges. The optimal threshold was determined by iterating across cost threshold values and selecting the threshold with maximized joint difference of global efficiency from lattices and local efficiency from random graphs. This approach maximizes small-world properties of the network [42] and yielded an optimal cost threshold of 0.2 for our dataset. Cost thresholds ensure networks of equal size as the same number of edges are retained (i.e., in our case only the strongest 20% of connections were retained in each subject). We extracted the global efficiency and clustering coefficient as network efficiency metrics [42–45]. The global efficiency is the inverse of average shortest paths between nodes. At the network level, it is considered to reflect communication efficiency (i.e., how fast the information circulates between the nodes of the networks). The clustering coefficient describes the mean probability that the nodes to which a node is connected are also connected to each other. At the network level, the clustering coefficient reflects the averaged coefficient across all the ROIs included in that network. High mean clustering is considered to be a measure of high resilience to change of the network.

We performed categorical analyses to compare HC with all patients, and with currently depressed and remitted patients separately. To examine the specific effects of PmD, we also compared healthy participants to both currently depressed and remitted patients with and without PmD, respectively. Then, we compared the PmD groups with each other. Finally, we compared the PmD groups between depressive states. We included regressors to account for the MR hardware and software changes, mFD, age, and sex in all analyses. We considered differences with q-FDR < 0.05 on cluster level to be significant. In addition, we compared global efficiency and clustering coefficient between groups. Furthermore, we performed dimensional analyses in patients with MDD to explore the association between symptom severity or outcome (as assessed by HAMD or GAF) and resting-state BOLD ROI-to-ROI connectivity within the cerebral motor network. Finally, a structural analysis has been performed using gray matter density of each of the ROIs, which is presented in Supplementary material A.

## Results

### Clinical and demographic findings

Age was not significantly different between the currently depressed patients and the other groups, but remitted patients were slightly older than healthy participants. Each group was approximately two-thirds female, whilst education did not differ between groups. As expected and partially per the criteria for group allocation, currently depressed participants had higher depression severity than the other groups and remitted participants showed higher depression severity than healthy participants.

### Clinical presentation of psychomotor disturbance

Proportions of patients with PmD and characteristics of PmD groups are presented in Table 2. Among the remitted patients, only two had concurrent PmR and PmA. Notably, depression severity did not differ between PmD groups in currently depressed or remitted patients, but participants with PmD had poorer global functioning (GAF) than those without.

Due to their small sample size, the sub-analyses dedicated to the patients presenting concurrent PmR and PmA and the comparisons between PmR and PmA in the remitted patients are presented in Supplementary material B.

### Functional connectivity at rest in the motor network

**Fig. 1:**
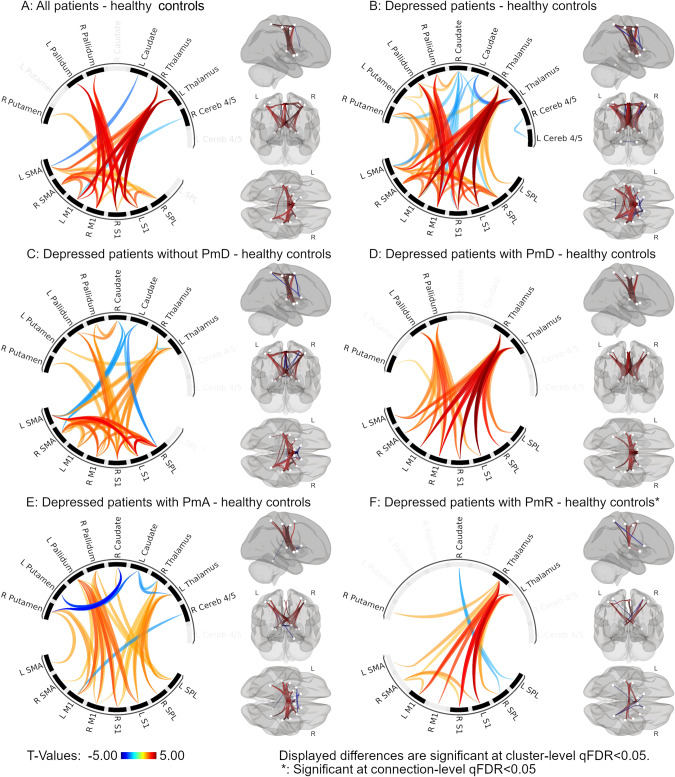
Red depicts connections with higher connectivity in patients, blue higher connectivity in controls. Panels depict differences between patient groups and healthy controls: **A** all patients; **B** depressed patients; **C** depressed patients without psychomotor disturbance; **D** depressed patients with psychomotor disturbance; **E** depressed patients with psychomotor agitation; **F** depressed patients with psychomotor retardation. All displayed differences have q-FDR < 0.05. Analysis adjusted for age, sex, mean framewise displacement, and scanner software and hardware changes.

ROI-to-ROI comparisons in the motor network between healthy participants and all patients showed alterations in the cerebral motor network with increased thalamo-cortical, pallidum-primary sensorimotor cortex, and cortico-cortical connectivity (Fig. 1A). However, subsequent analyses comparing healthy with remitted and currently depressed patients separately only showed differences between the currently depressed patients and healthy participants (Fig. 1B). There were no differences between remitted patients and healthy participants, nor between currently depressed and remitted patients.

Next, the comparison of currently depressed patients with PmD and HC showed higher thalamo-cortical and pallido-cortical connectivity in depressed patients with PmD (Fig. 1D). Investigating specific types of PmD, currently depressed patients with PmA had higher thalamo-cortical and pallido-cortical, but lower subcortical (thalamus-caudate, putamen-caudate) connectivity than HC (Fig. 1E). Currently depressed patients with PmR had higher thalamo-cortical and lower caudate-SPL connectivity only at ROI-level FDR correction instead of cluster-level FDR correction (Fig. 1F). Comparing currently depressed patients without PmD and HC, we observed differences that were very similar to those in the comparison of all currently depressed patients and HC, including higher thalamo-cortical, pallido-cortical, and cortico-cortical connectivity, as well as lower cortico-caudate connectivity in currently depressed patients without PmD (Fig. 1C). Statistical details are presented in Supplementary material C.

Comparison between remitted patients with and without PmD and HC showed no differences in connectivity in the cerebral motor system. We found no differences in functional connectivity in the cerebral motor network between currently depressed patients with or without PmD, nor between patients with PmR and those with PmA. In sum, currently depressed with PmD, PmA or PmR differed from HC in cortico-subcortical functional connectivity in the motor circuit.

Reproduction of the analyses with scrubbing and with PmD-affected healthy participants showed no substantially different results than the main analyses.

### Graph theory network metrics

**Fig. 2:**
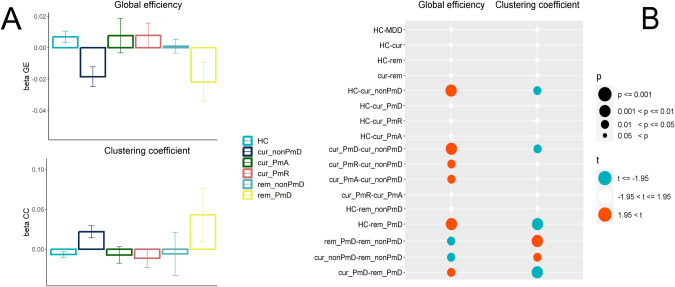
Graph theory of the motor network functional connectivity. **A** Effects for graph theoretical measures of the motor network per group (beta values and standard errors). **B** Between-group differences in global efficiency and clustering coefficient of the motor network. Effects adjusted for MR hardware and software changes, mean framewise displacement, age, and sex. HC healthy controls, cur currently depressed patients, rem remitted patients, PmD psychomotor disturbance, PmA psychomotor agitation, PmR psychomotor retardation.

Adjusted effects for graph theoretical measures per group and significant between-group differences are displayed in Fig. 2. No differences emerged between HC and all patients, HC and currently depressed or remitted patients separately, as well as between currently depressed and remitted patients.

Similarly, there was no difference between current PmD, current PmA, or current PmR compared to HC. However, lack of PmD in current MDD was associated with lower efficiency in the motor network: currently depressed patients without PmD had lower global efficiency and higher clustering coefficient than both HC or currently depressed with PmD. Similarly, currently depressed patients with either PmR or PmA had higher global efficiency than those without PmD.

Among the remitted patients, PmD was linked to aberrant motor network metrics: Patients with PmD or PmR had lower global efficiency and higher clustering coefficients compared to HC or remitted patients without PmD. In turn, remitted patients without PmD did not show any differences compared to HC.

When we compared pairs of PmD subgroups between currently depressed and remitted patients, we found that currently depressed patients with PmD or PmR had higher global efficiency and lower clustering than their remitted counterparts. But the effect was reversed for patients without motor symptoms: currently depressed patients without PmD had lower global efficiency and higher clustering coefficient in the motor network compared to remitted patients without PmD. Detailed statistics are presented in Supplementary material C.

### Dimensional analyses of symptoms and functioning

**Fig. 3:**
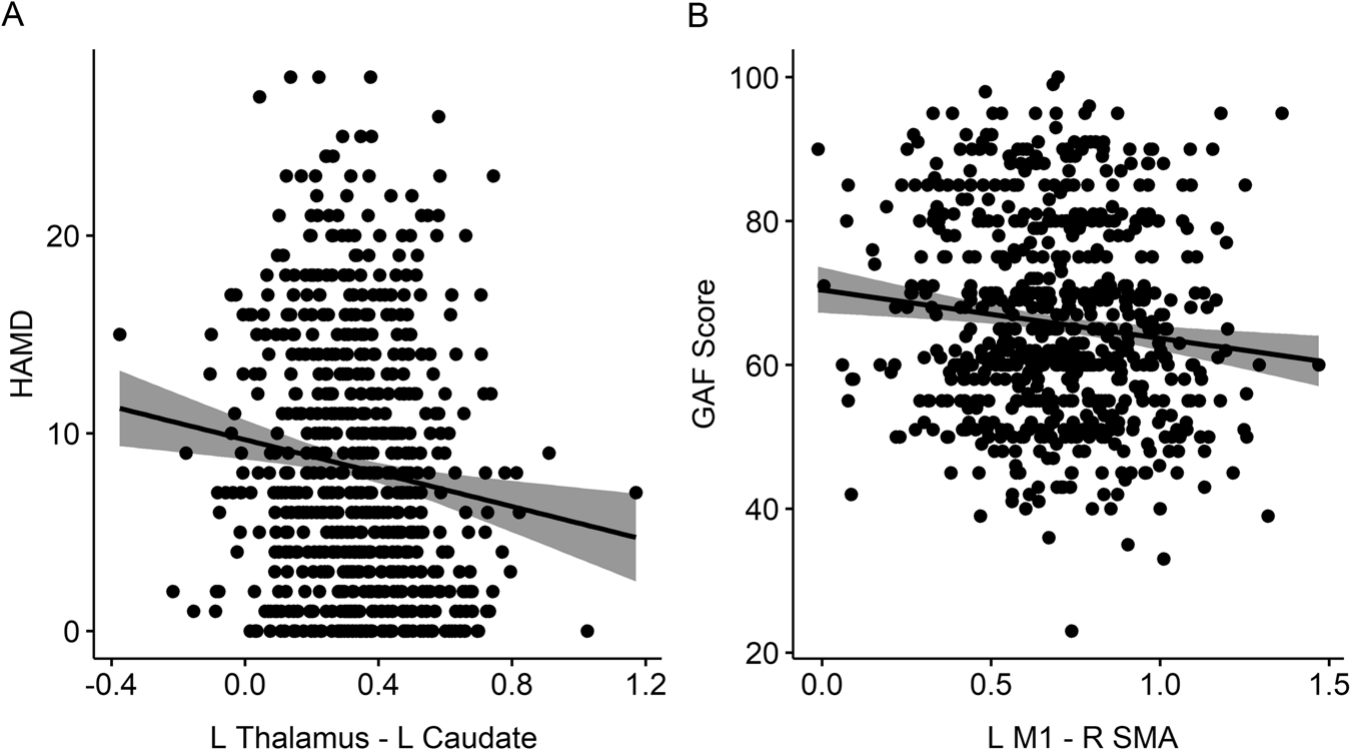
Dimensional analyses of symptoms and functioning. **A** Shows a scatter plot of the significant association between HAMD and the functional connectivity between L thalamus and L caudate. **B** Shows a scatter plot of the significant association between GAF and the functional connectivity between L M1 and L SMA. Shaded areas reflect the confidence intervals of the regression lines. HAMD Hamilton Depression Rating Scale, L left, R right.

Depression severity as measured with HAMD was associated with a bilateral decrease of thalamus-caudate functional connectivity (Fig. 3A, Supplementary material C). Moreover, impaired global functioning was associated with decreased bilateral connectivity in the cerebellum (lobules 4−5) and an increased cortico-cortical connectivity between bilateral post/precentral gyri and the bilateral SMA (Fig. 3B, Supplementary material C). Among patients with MDD, depression severity was not linked to graph theory metrics. However, impaired global functioning was associated with lower global efficiency and a higher clustering coefficient in the motor network (Supplementary material C).

### Structural analyses

No group difference was detected in the GMD of any ROI. In addition, we could not observe any significant association between the GMD of each ROI and clinical ratings (GAF and HAMD scores) following FDR correction for 18 ROIs (all tau >0.05, all *p*_*FDR*_ > 0.2)). These results are presented in Supplementary material A.

## Discussion

The current study sought to examine the neural correlates of PmD in MDD using resting-state functional connectivity and graph theoretical measures in a large cohort (1519 participants from the MACS dataset [27]). In line with our hypotheses, currently depressed patients with PmD had specific resting-state connectivity alterations in the motor circuit compared to HC. Results varied by the type of PmD. Subjects with PmR had higher connectivity in thalamo-cortical connections, while subjects with PmA had higher connectivity in pallido-cortical connections. None of these functional connectivity alterations were observed in remitted patients. Additionally, network characteristics differed between patients with and those without PmD and these differences had opposite directions in currently depressed and remitted patients.

### Clinical presentation of psychomotor disturbance in MDD

More than half of the currently depressed and one of six individuals with remitted MDD had PmD, demonstrating the importance of PMD in current and remitted MDD [13, 15]. These numbers (55% and 16%) of PmD frequencies are lower than those of previous studies reporting PMD in 70−75% of severely depressed patients [9–13]. This discrepancy could be explained by several specificities of the current sample. First, the cohort was not designed to assess motor disturbances with specific instruments. In addition, the average depression severity in the currently depressed group was mild to moderate, indicating that our sample did not include a large proportion of cases with severe depression, in whom PmD is more frequent [46–49]. We did not find that higher depression severity was associated with PmD in the currently depressed patients [50, 51]. However, both remitted and currently depressed patients with PmD had lower global functioning as measured by the GAF than the groups without PmD. Therefore, although PmD was unrelated to higher depression severity in our sample, the presence of PmD was associated with more impairment, even in remitted patients.

### Functional connectivity alterations within the motor network in patients with MDD and PmD

The cerebral motor system is mainly in an inhibitory mode: only to allow releasing specific movements, disinhibition is used. Ongoing movement is primarily controlled by inhibitory action, for example via the SMA-subthalamic nucleus hyperdirect pathway to stop ongoing movement rapidly [52, 53]. The categorical resting-state functional connectivity analyses revealed alterations of the cerebral motor network in all currently depressed patients, even those without PmD. In line with the current literature [54], depression is associated with altered connectivity strength in the motor cortico-basal ganglia circuit and in particular within the thalamo-cortical and pallido-cortical connections. The classical model of the motor cortico-basal ganglia circuit consists of top-down inputs from (pre-)motor cortical areas to the striatum, bottom-up outputs from thalamus back to the cortical origins of the input, and the subcortical core circuit that includes the globus pallidus, subthalamic nucleus and substantia nigra [55, 56]. Furthermore, there are a number of cortico-cortical connections between cerebral motor areas of the prefrontal and parietal cortex [57]. The current understanding is that cortico-cortical connections may modulate top-down and bottom-up signaling in the motor circuit, giving rise to psychomotor phenomena [26]. In line with this current top-down/bottum-up hypothesis and despite the lack of effective connectivity analyses, we suggest the following interpretation of the present results: Currently depressed subjects had stronger connectivity between M1 and pallidum, thalamus and motor cortex, as well as more cortico-cortical connectivity, while connectivity between SMA and caudate was reduced compared to HC, which could reflect a decreased input from SMA to caudate. Furthermore, the observed lower cortico-striatum connectivity could suggest that depression without PmD specifically has reduced motor top-down inputs to the striatum. Consequently, the motor-associated bottom-up outputs from the thalamus would be altered. In short, we suggest that reduced (pre-)motor cortical inputs cause altered thalamic outputs. Concurrently, we observed strongly increased cortico-cortical connectivity within the motor network, which might compensate for aberrant bottom-up signaling in MDD without PmD. In contrast, currently depressed patients with PmD did not show alterations in cortico-caudate or cortico-cortical connections within the motor network, while they had similarily increased connectivity in thalamo-cortical and pallido-cortical projections compared to controls. This finding supports the hypothesis of a compensatory increase in cortico-cortical connectivity within the motor network that could prevent PmD in MDD.

The associations of resting state connectivity with symptoms and community functioning also speak towards compensatory connectivity changes in MDD. Specifically, patients with lower global functioning showed increased cortico-cortical connectivity in premotor and motor cortices. In addition, alterations in thalamus-caudate resting-state functional connectivity are associated with increased depression severity. These results emphasize the important role of the motor network in depression severity and functional outcome [13]. For example, PmR is related to familial aggregation of depression, treatment effects, symptom severity as well as poor reward learning [50, 58–61].

### Graph theoretical alterations of the motor network

Comparison of HC and currently depressed patients on network metrics revealed no differences in topology of the motor network with one exception: The subgroup without PmD had lower global efficiency and higher clustering than both HC and patients with PmD. This suggests either that current depression has a perturbing effect on the network that is compensated in patients with PmD, or that compensatory changes that prevent PmD may perturb the network in patients without PmD. The latter of these two interpretations aligns with the results of our classical functional connectivity analysis. Remitted patients showed inverse effects: Remitted patients with PmD had lower global efficiency and higher clustering coefficient than HC and patients without PmD, indicating that some motor network alterations persist in patients with PmD during remission. As there were no differences between remitted patients with PmD and the other groups in the classical functional connectivity analyses, these alterations are likely very subtle on the single connection level. The lack of compensatory changes in current depression with PmD and persisting changes in remitted depression with PmD point towards reduced adaptability in PmD.

Moreover, our data suggests largely preserved polarity of functional connectivity of the motor network across groups, while the aberrant magnitude of motor network connectivity might drive PmD. Given that a number of psychiatric disorders including MDD are associated with an excitatory/inhibitory imbalance in cerebral circuits [62], this might be specifically true for the motor circuit. Other studies on resting-state connectivity found similar alterations in PmD across different populations (bipolar disorder and schizophrenia) [63, 64], suggesting transdiagnostic pathways. These results support NIMH’s RDoC framework, which calls for multidimensional and transdiagnostic examination of sensorimotor constructs [13]. Conversely, Cantisani et al. reported differences in the associations of cerebral blood flow and physical activity between unipolar and bipolar depressed patients, challenging the transdiagnostic approach [65]. As we hypothesized, these functional connectivity alterations associated with PmD seem to depend on depression states. In fact, no resting-state functional connectivity alterations of the motor network were found in the classical ROI-to-ROI analysis in remitted patients, even in those with PmD and graph metrics were inversed between mood states. Thus, the rs-fMRI alterations in the motor circuit may be most prominent during acute illness and persist into remission in a more subtle form only in a subpopulation of patients with the most robust network alterations.

Within PmD, findings suggest distinct changes of motor network connectivity in PmR and PmA, albeit different than hypothesized by Northoff and colleagues [66]. PmR was characterized by higher thalamo-cortical connectivity without changes in pallido-cortical connectivity. As PmR is hypokinetic, the observed increase in thalamo-cortical activity is likely net inhibitory. Specifically, the alterations were more pronounced in the connections involving primary motor and sensory cortices but were also present in the connections with SMA. These results parallel previous work demonstrating that PmR in MDD is linked to altered structural connectivity in motor pathways [22, 23], cerebral blood flow in M1 [25], and task-based connectivity between SMA and basal ganglia [67]. Similarly, increased thalamo-cortical connectivity in schizophrenia was linked to hypokinetic motor abnormalities [68]. In contrast, PmA was characterized by prominent higher pallido-cortical connectivity focusing on M1 and S1. While higher thalamocortical connectivity was also present in PmA, it was restricted to parietal cortices. However, our findings argue against a simple transdiagnostic continuum in which PmR results from reduced thalamo-cortical connectivity and PmA from increased thalamo-cortical connectivity as suggested by Northoff and colleagues [66]. Albeit very useful, their model requires more testing and updating. Finally, the data of the current study may guide efforts to apply neuromodulation to aberrant motor networks in MDD. Both M1 and SMA could be valuable targets for transcranial magnetic stimulation. A prior study found that inhibitory rTMS on the SMA ameliorated PmR in a transdiagnostic sample including MDD [69].

### Limitations

The present study has some limitations. As mentioned above, this study was not designed to assess PmD specifically. Therefore, a relevant number of patients with subtle PmD might have been considered as having no PmD. Our sample showed moderate total depression and PmD severity and a more severely depressed population might include a larger proportion of patients with PmD. Thus, generalizability to more severely depressed patients may be limited and our results warrant replication in such a sample. Even if the current study is providing a large overview of the brain alterations associated with PmD in MDD, the understanding of these complex conditions would benefit from adding further imaging modalities, such as structural connectivity, effective connectivity, task-based connectivity and activations. Finally, to capture the trait vs. state effects, longitudinal analyses would be required in a group of patients with PmD.

## Conclusion

The present study is the first one to provide an exploration of the neural correlates of PmD in MDD using classical resting-state functional connectivity as well as network measures based on graph theory. PmA and PmR showed specific connectivity signatures within the motor network while we found evidence for compensatory increased cortico-cortical connectivity that may prevent PmD in current depression. This compensatory connectivity change disturbs network topology. In contrast, remitted patients with PmD show altered network topology of the motor network in the absence of single connection alterations, suggesting reduced adaptability of the motor network in patients with PmD.

### Supplementary information

Supplementary material

## Data Availability

The data that support the findings of the current manuscript are available from the corresponding authors, SL and FW, upon reasonable request.
