# Supplementary material for: The neural signature of psychomotor disturbance in depression
Source: Mol Psychiatry. 2023 Dec 1;29(2):317–26. doi: 10.1038/s41380-023-02327-1 (PMC11116107; doi:10.1038/s41380-023-02327-1)
Supplement: Supplementary file 1 — Supplementary material [file 41380_2023_2327_MOESM1_ESM.docx]

# **Supplementary materials to:**

# **The neural signature of psychomotor disturbance in depression**

Florian Wüthrich^1,2*^, Stephanie Lefebvre^1*^, Vijay A Mittal^3-7^, Stewart A. Shankman^3,4^, Nina Alexander^8^, Katharina Brosch^8,9^, Kira Elisa Flinkenflügel^10^, Janik Goltermann^10,^ Dominik Grotegerd^10,^ Tim Hahn^10^, Hamidreza Jamalabadi^8^, Andreas Jansen^8,9,11^, Elisabeth J. Leehr^10^, Susanne Meinert^10,12^, Igor Nenadic^8,9^, Robert Nitsch^12^, Frederike Stein^8,9^, Benjamin Straube^8,9^, Lea Teutenberg^8,9^, Katharina Thiel^10,^ Florian Thomas-Odenthal^8,9^, Paula Usemann^8,9^, Alexandra Winter^10^, Udo Dannlowski^10^, Tilo Kircher^8,9^, Sebastian Walther^1^

* contributed equally, corresponding authors

^1^ Translational Research Center, University Hospital of Psychiatry and Psychotherapy, University of Bern, Switzerland

^2^ Graduate School of Health Science, University of Bern, Switzerland

^3^ Northwestern University, Department of Psychiatry and Behavioral Sciences, Chicago, IL, USA.

^4^ Northwestern University, Department of Psychology, Evanston, IL, USA

^5^ Northwestern University, Institute for Innovations in Developmental Sciences, Evanston/Chicago, IL, USA

^6^ Northwestern University, Institute for Policy Research, Evanston, IL, USA

^7^ Northwestern University, Medical Social Sciences, Chicago, IL, USA

^8^ Department of Psychiatry and Psychotherapy, University of Marburg, Germany

^9^ Center for Mind, Brain and Behavior (CMBB), University of Marburg, Germany

^10^ Institute for Translational Psychiatry, University of Münster, Germany

^11^ Core-Facility Brainimaging, Faculty of Medicine, University of Marburg, Germany

^12^ Institute for Translational Neuroscience, University of Münster, Germany

**Table of content**

**Supplementary A: Structural Analyses**

Grey Mater Density processing
 Results

Supplementary Figure A1 : Grey matter density of the 18 ROIs for each group.

Supplementary Figure A2 : Grey matter density of the 18 ROIs association with GAF.

Supplementary Figure A3 : Grey matter density of the 18 ROIs association with HAMD

**Supplementary B: Analyses dedicated to the *concurrent* PmA and PmR in currently depressed population and to the PmA and PmR distinction in the remitted population.**

Supplementary Table B1: Between-group differences in graph measures for patients with concurrent PmA and PmR

Supplementary Figure B1 Extension of the Figure 2 of the main text

**Supplementary C: Tables for functional connectivity**

Supplementary Table C1: ROI-to-ROI categorical analysis statistic table

Supplementary Table C2: Graph theory functional connectivity categorical analysis statistic table

Supplementary Table C3: ROI-to-ROI dimensional analysis statistic table

Supplementary Table C4: Graph theory dimensional analysis statistic table

**Supplementary Material A**

*Grey Mater Density processing*

To analyse the grey matter density (GMD), we performed VBM preprocessing and analysis, with the CAT12 VBM algorithm (CAT12 toolbox (http://www.neuro.uni-jena.de/cat/) in SPM12 (Version 7771, Welcome Trust, London, UK. https://www.fil.ion.ucl.ac.uk/spm)). The T1-3D images were segmented into grey matter (GM), white matter, and cerebrospinal fluid maps, then, the native GM maps were modulated, normalized to the MNI template, and smoothed using a 6 mm FHWM Gaussian kernel. We extracted the mean GMD value from each of the ROIs included in the ROI-to-ROI functional connectivity analyses.
To examine the association between GMD in the motor network and PmD in MDD, we performed categorical and dimensional analyses. For the categorical analyses, we ran ANCOVAs for each ROI to compare the GMD between the different groups (age, sex, MR-hardware and software changes as well as TIV (total intracranial volume) were used as controlling variables). For the dimensional analyses, we performed Kendall Tau partial correlations (Age, sex, MR-hardware and software changes, and TIV) between the GMD of each ROIs and symptom severity (GAF and HAMD scores). Age, sex, MR-hardware and software changes as well as TIV (total intracranial volume) were used as controlling variables.

*Results*

We did not find any difference in the GMD of each ROIs between the different groups (Supplementary Figure A1). We also did not observe any significant association (FDR corrected for the 18 regions) between the GMD of each ROIs and the symptom severity (GAF and HAMD scores (all tau >0.05, all p*_FDR_* >0.2)) (Supplementary Figure A2 and A3).


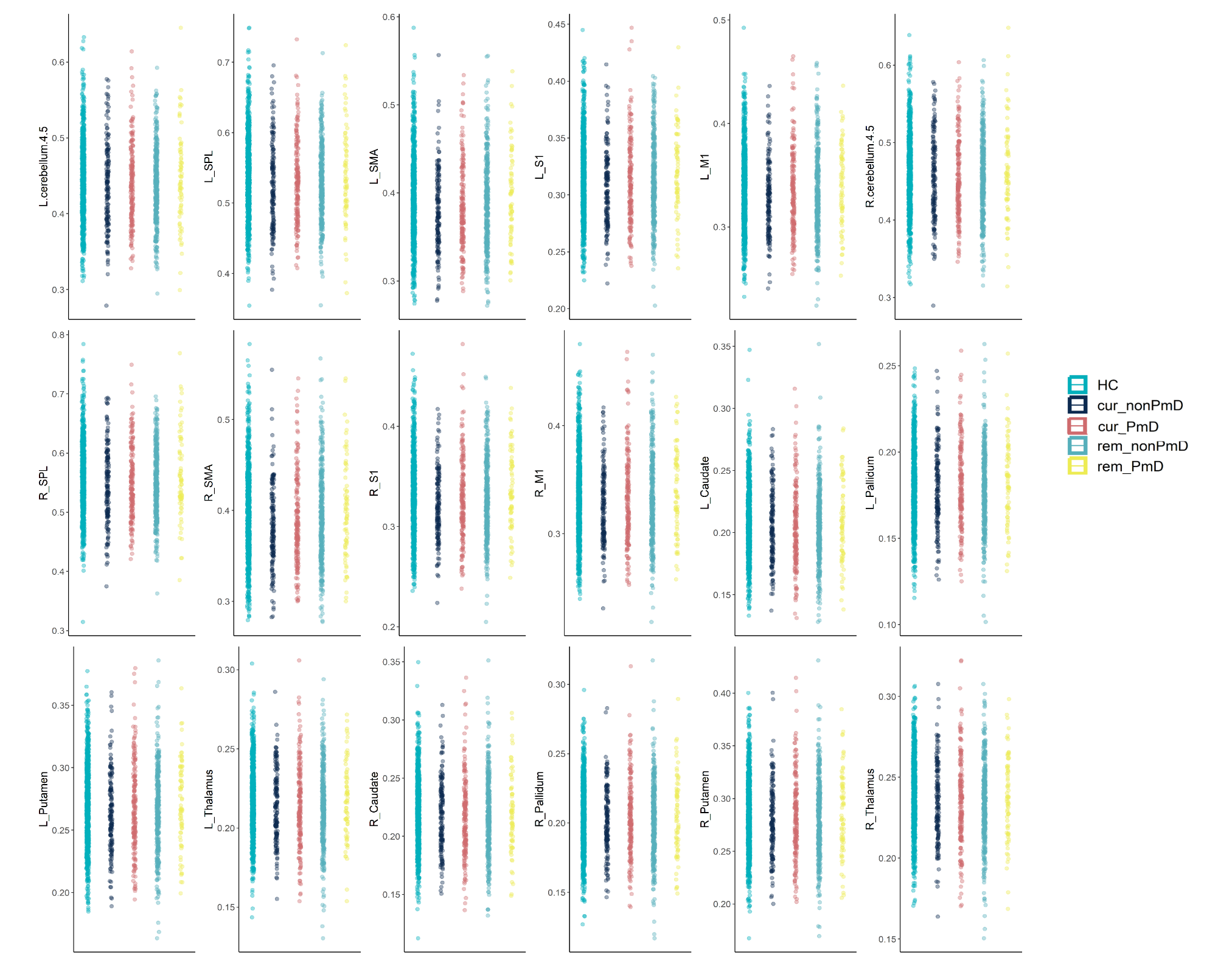


**Supplementary Figure A1:**

**Grey matter density of the 18 ROIs for each group.**No significant differences were observed between the groups.


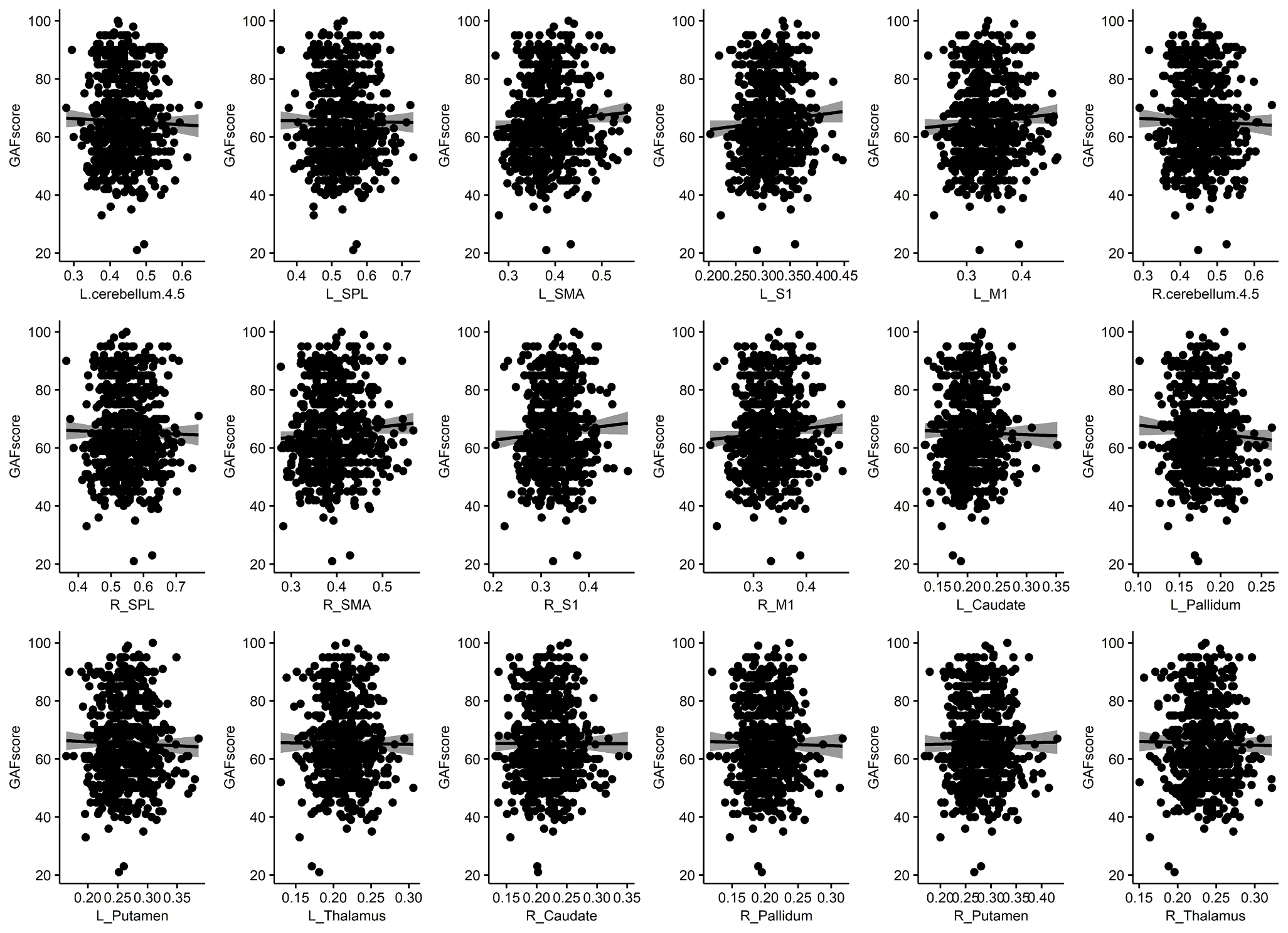


**Supplementary Figure A2:**

**Grey matter density of the 18 ROIs association with GAF.**No significant associations were observed.

**
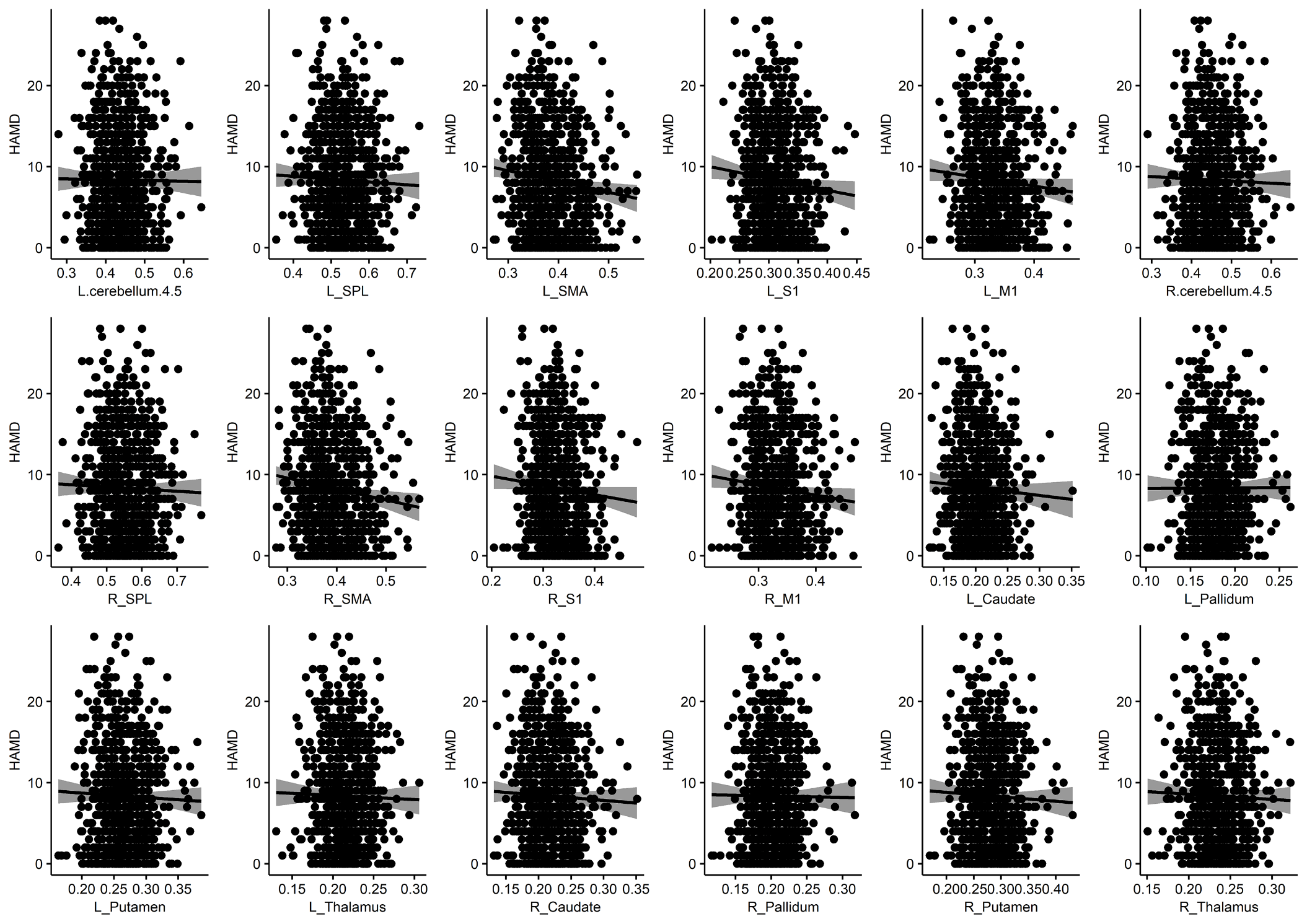
**

**Supplementary Figure A3:**

**Grey matter density of the 18 ROIs association with GAF.**No significant associations were observed.

**Supplementary Material B: Analyses dedicated to the *concurrent* PmA and PmR in currently depressed population and to the PmA and PmR distinction in the remitted population.**

## **Functional Connectivity at rest in the motor network**

Currently depressed participants with *concurrent* PmA and PmR showed no differences compared with HC.

## **Graph theory network metrics**

| **Table B1 Between-group differences in graph measures for patients with concurrent PmA and PmR** | | | | | | | | |
| --- | --- | --- | --- | --- | --- | --- | --- | --- |
|  | Global Efficiency | | | | Clustering Coefficient | | | |
| Contrast | Δβ | t-value | p-value | pFDR | Δβ | t-value | p-value | pFDR |
| HC-curPmM | 0.010 | 0.610 | 0.540 | 0.784 | 0.010 | 0.340 | 0.730 | 0.840 |
| curPmM-curnonPmD | -0.010 | -0.910 | 0.360 | 0.630 | 0.030 | 1.300 | 0.190 | 0.429 |
| curPmM-curPmA | -0.010 | -0.740 | 0.461 | 0.717 | <0.001 | -0.110 | 0.911 | 0.945 |
| curPmM-curPmR | -0.010 | -0.830 | 0.407 | 0.670 | <0.001 | 0.020 | 0.980 | 0.980 |
| Differences in graph measures adjusted for age, sex, MR-hardware and software changes, and in-scanner movement. *Italics*: significant at uncorrected p<0.05*.* **Bold:** significant at FDR-corrected q<0.05.  HC: healthy controls; MDD: people with major depressive disorder diagnosis; cur: currently depressed individuals; rem: remitted individuals; nonPmD: without psychomotor disturbance; PmD: with psychomotor disturbance; PmR: with psychomotor retardation; PmA: with psychomotor agitation; PmM: concurrent psychomotor retardation and agitation. | | | | | | | | |


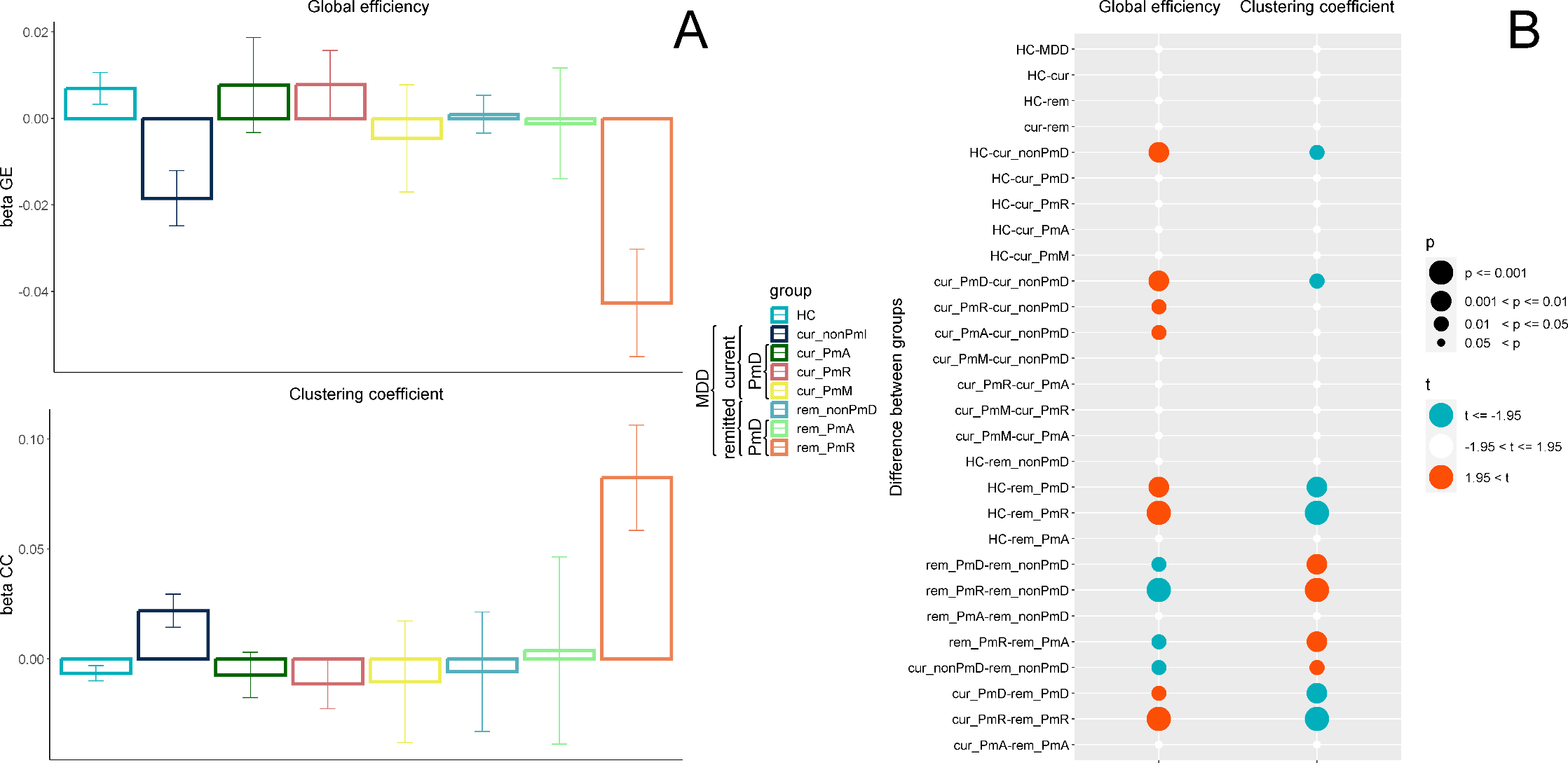


**Supplementary Figure B1: Extension of the Figure 2** of the main paper including sub-analyses dedicated to the patients presenting both PmR and PmA and the ones related to the distinction between PmR and PmA in the remitted population. A: Effects for graph theoretical measures of the motor network per group (beta values and standard errors). B: Between-group differences in global efficiency and clustering coefficient of the motor network. Effects adjusted for MR hardware and software changes, mean framewise displacement, age, and sex. HC: healthy controls, cur: currently depressed patients, rem: remitted patients, PmD: psychomotor disturbance, PmA: psychomotor agitation, PmR: psychomotor retardation, PmM: concurrent psychomotor agitation and retardation.

**Supplementary Material C**

| **Table C1: Group comparisons of functional connectivity for selected contrasts** | | | | | |
| --- | --- | --- | --- | --- | --- |
| **MDD-HC** |  |  | **Cur-HC** |  |  |
| Connection | Statistic | p-value | Connection | Statistic | p-value |
| Cortico-Cortical Clusters | F(_4,1508)_ = 4.40 | 0.002 | Cortico-Cortical Clusters | F(_4,1507)_ = 6.33 | 0.000 |
| L S1-R SPL | T(1511) = 3.03 | 0.002 | L S1-R SPL | T(1510) = 3.25 | 0.001 |
| R SMA-R M1 | T(1511) = 3.47 | 0.001 | R S1-R SPL | T(1510) = 3.18 | 0.001 |
| R SMA-L S1 | T(1511) = 3.19 | 0.001 | R SMA-L S1 | T(1510) = 3.31 | 0.001 |
| R SMA-L M1 | T(1511) = 2.82 | 0.005 | R SMA-R M1 | T(1510) = 3.20 | 0.001 |
| L SMA-L S1 | T(1511) = 2.47 | 0.014 | R M1-R SPL | T(1510) = 2.73 | 0.006 |
| R SMA-R S1 | T(1511) = 2.35 | 0.019 | R SMA-L M1 | T(1510) = 2.83 | 0.005 |
| L SMA-R M1 | T(1511) = 2.26 | 0.023 | L SMA-L S1 | T(1510) = 2.56 | 0.011 |
| L SMA-R SMA | T(1511) = 2.20 | 0.028 | L SMA-R SMA | T(1510) = 2.37 | 0.018 |
| R M1-R SPL | T(1511) = 2.11 | 0.035 | L SMA-R M1 | T(1510) = 2.27 | 0.023 |
| R S1-R SPL | T(1511) = 2.18 | 0.029 | R SMA-R S1 | T(1510) = 2.25 | 0.025 |
| L SMA-R SPL | T(1511) = 2.08 | 0.038 | R SMA-R SPL | T(1510) = 2.17 | 0.029 |
| R SMA-R SPL | T(1511) = 1.99 | 0.046 | L M1-R SPL | T(1510) = 2.26 | 0.024 |
|  |  |  | L SMA-R SPL | T(1510) = 2.07 | 0.038 |
|  | | | | | |
| Cortical-Subcortical Clusters | F(_4,1508)_ = 5.73 | 0.000 | Cortical-Subcortical Clusters | F(_4,1507)_ = 6.30 | 0.000 |
| R S1-L Thalamus | T(1511) = 4.56 | 0.000 | L S1-R Thalamus | T(1510) = 4.85 | 0.000 |
| R S1-R Thalamus | T(1511) = 4.40 | 0.000 | R S1-R Thalamus | T(1510) = 4.59 | 0.000 |
| L S1-R Thalamus | T(1511) = 4.37 | 0.000 | R S1-L Thalamus | T(1510) = 4.47 | 0.000 |
| L S1-L Thalamus | T(1511) = 4.24 | 0.000 | L S1-L Thalamus | T(1510) = 4.44 | 0.000 |
| L M1-L Thalamus | T(1511) = 4.25 | 0.000 | L M1-R Thalamus | T(1510) = 4.44 | 0.000 |
| R S1-L Pallidum | T(1511) = 3.95 | 0.000 | L M1-L Thalamus | T(1510) = 4.38 | 0.000 |
| L S1-L Pallidum | T(1511) = 3.90 | 0.000 | R M1-L Pallidum | T(1510) = 4.25 | 0.000 |
| R M1-L Pallidum | T(1511) = 4.04 | 0.000 | R M1-L Thalamus | T(1510) = 4.00 | 0.000 |
| R M1-L Thalamus | T(1511) = 3.96 | 0.000 | R M1-R Pallidum | T(1510) = 3.98 | 0.000 |
| L M1-R Thalamus | T(1511) = 3.88 | 0.000 | R M1-R Thalamus | T(1510) = 3.83 | 0.000 |
| L M1-L Pallidum | T(1511) = 3.80 | 0.000 | R S1-L Pallidum | T(1510) = 3.57 | 0.000 |
| R S1-R Pallidum | T(1511) = 3.58 | 0.000 | R S1-R Pallidum | T(1510) = 3.49 | 0.000 |
| R M1-R Thalamus | T(1511) = 3.57 | 0.000 | L S1-R Pallidum | T(1510) = 3.41 | 0.001 |
| R M1-R Pallidum | T(1511) = 3.27 | 0.001 | L SMA-L Thalamus | T(1510) = 3.66 | 0.000 |
| L S1-R Pallidum | T(1511) = 3.27 | 0.001 | L M1-L Pallidum | T(1510) = 3.32 | 0.001 |
| L SMA-L Thalamus | T(1511) = 3.11 | 0.002 | L S1-L Pallidum | T(1510) = 3.07 | 0.002 |
| L SMA-R Thalamus | T(1511) = 2.87 | 0.004 | L SMA-R Thalamus | T(1510) = 3.37 | 0.001 |
| L SMA-L Caudate | T(1511) = -2.77 | 0.006 | R SMA-L Thalamus | T(1510) = 3.09 | 0.002 |
| L S1-R Putamen | T(1511) = 2.26 | 0.024 | R M1-R Putamen | T(1510) = 2.80 | 0.005 |
| R SMA-L Thalamus | T(1511) = 2.45 | 0.015 | L M1-R Pallidum | T(1510) = 2.90 | 0.004 |
| R M1-R Putamen | T(1511) = 2.06 | 0.039 | L S1-R Putamen | T(1510) = 2.50 | 0.013 |
| L M1-R Pallidum | T(1511) = 2.18 | 0.029 | L SMA-L Pallidum | T(1510) = 2.66 | 0.008 |
| L M1-R Cerebellum 4/5 | T(1511) = -2.13 | 0.034 | L SMA-R Pallidum | T(1510) = 2.48 | 0.013 |
|  | | | L SMA-L Caudate | T(1510) = -2.38 | 0.018 |
|  |  |  | L SMA-R Caudate | T(1510) = -2.37 | 0.018 |
|  |  |  | L SMA-R Putamen | T(1510) = 2.31 | 0.021 |
|  |  |  | R SMA-L Pallidum | T(1510) = 2.53 | 0.012 |
|  |  |  | R SMA-R Pallidum | T(1510) = 2.46 | 0.014 |
|  |  |  | L M1-R Putamen | T(1510) = 2.27 | 0.023 |
|  |  |  | L M1-R Caudate | T(1510) = -2.21 | 0.027 |
|  |  |  | R SMA-R Thalamus | T(1510) = 2.12 | 0.035 |
|  |  |  | R M1-R Caudate | T(1510) = -2.14 | 0.032 |
|  |  |  | R M1-L Putamen | T(1510) = 2.05 | 0.040 |
|  |  |  | R SPL-R Thalamus | T(1510) = 2.17 | 0.030 |
|  | | | R SPL-L Caudate | T(1510) = -2.06 | 0.039 |
|  |  |  | R SPL-L Thalamus | T(1510) = 2.06 | 0.039 |
|  |  |  | R S1-R Caudate | T(1510) = -2.07 | 0.039 |
|  |  |  | L SPL-R Thalamus | T(1510) = 2.49 | 0.013 |
|  |  |  | L SPL-L Thalamus | T(1510) = 2.05 | 0.040 |
|  |  |  |  | | |
|  |  |  | Subcortical-Subcortical Cluster | F(4,1507) = 4.43 | 0.001 |
|  |  |  | L Thalamus-L Caudate | T(1510) = -2.88 | 0.004 |
|  |  |  | R Thalamus-R Putamen | T(1510) = 2.12 | 0.034 |
|  |  |  | L Thalamus-R Caudate | T(1510) = -2.06 | 0.039 |
|  |  |  | L Pallidum-L Putamen | T(1510) = 2.12 | 0.034 |
|  |  |  | R Caudate-R Pallidum | T(1510) = 2.45 | 0.014 |
|  |  |  | R Caudate-L Pallidum | T(1510) = 2.14 | 0.033 |
|  |  |  | R Caudate-R Putamen | T(1510) = -2.13 | 0.034 |
|  |  |  | L Cerebellum 4/5-R Cerebellum 4/5 | T(1510) = -2.22 | 0.027 |
|  | | | | | |
| **Cur without PmD-HC** |  |  | **Cur with PmD** |  |  |
| Connection | Statistic | p-value | Connection | Statistic | p-value |
| Cortico-Cortical Clusters | F(_4,1507)_ = 6.57 | 0.000 |  | | |
| L S1-R SPL | T(1510) = 3.93 | 0.000 |  |  |  |
| L SMA-L S1 | T(1510) = 3.81 | 0.000 |  |  |  |
| L SMA-R SPL | T(1510) = 3.30 | 0.001 |  |  |  |
| R S1-R SPL | T(1510) = 3.35 | 0.001 |  |  |  |
| L SMA-R M1 | T(1510) = 2.85 | 0.004 |  |  |  |
| L SMA-R S1 | T(1510) = 2.85 | 0.005 |  |  |  |
| R M1-R SPL | T(1510) = 2.79 | 0.005 |  |  |  |
| R SMA-L S1 | T(1510) = 3.15 | 0.002 |  |  |  |
| R SMA-R M1 | T(1510) = 3.00 | 0.003 |  |  |  |
| L SMA-L M1 | T(1510) = 2.50 | 0.013 |  |  |  |
| L SMA-R SMA | T(1510) = 2.22 | 0.027 |  |  |  |
| R SMA-R SPL | T(1510) = 2.63 | 0.009 |  |  |  |
| L M1-R SPL | T(1510) = 2.44 | 0.015 |  |  |  |
| R SMA-R S1 | T(1510) = 2.24 | 0.025 |  |  |  |
| R SMA-L M1 | T(1510) = 2.12 | 0.034 |  |  |  |
|  | | | | | |
| Cortical-Subcortical Clusters | F(_4,1507)_ = 3.20 | 0.013 | Cortical-Subcortical Cluster | F(_4,1507)_ = 4.33 | 0.001 |
| R M1-L Pallidum | T(1510) = 2.86 | 0.004 | L S1-R Thalamus | T(1510) = 4.92 | 0.000 |
| R SPL-L Caudate | T(1510) = -2.52 | 0.012 | R S1-R Thalamus | T(1510) = 4.63 | 0.000 |
| R M1-L Thalamus | T(1510) = 2.44 | 0.015 | R S1-L Thalamus | T(1510) = 4.42 | 0.000 |
| R M1-R Pallidum | T(1510) = 2.43 | 0.015 | L S1-L Thalamus | T(1510) = 4.24 | 0.000 |
| L SMA-R Thalamus | T(1510) = 2.35 | 0.019 | L M1-R Thalamus | T(1510) = 4.16 | 0.000 |
| L SMA-R Caudate | T(1510) = -2.30 | 0.021 | L M1-L Thalamus | T(1510) = 4.07 | 0.000 |
| L SMA-L Thalamus | T(1510) = 2.25 | 0.025 | R M1-R Thalamus | T(1510) = 4.00 | 0.000 |
| L SMA-L Caudate | T(1510) = -2.25 | 0.025 | R M1-L Thalamus | T(1510) = 3.80 | 0.000 |
| L S1-L Thalamus | T(1510) = 2.45 | 0.014 | R M1-L Pallidum | T(1510) = 3.44 | 0.000 |
| L S1-R Thalamus | T(1510) = 2.37 | 0.018 | R M1-R Pallidum | T(1510) = 3.13 | 0.002 |
| L S1-L Pallidum | T(1510) = 2.31 | 0.021 | R SPL-R Thalamus | T(1510) = 3.50 | 0.001 |
| L S1-R Pallidum | T(1510) = 2.25 | 0.025 | R SPL-L Thalamus | T(1510) = 3.28 | 0.001 |
| R SPL-R Caudate | T(1510) = -2.19 | 0.029 | L M1-L Pallidum | T(1510) = 3.03 | 0.002 |
| L M1-L Thalamus | T(1510) = 2.63 | 0.009 | R S1-L Pallidum | T(1510) = 3.01 | 0.003 |
| L M1-R Thalamus | T(1510) = 2.60 | 0.009 | L SMA-L Thalamus | T(1510) = 3.27 | 0.001 |
| R M1-R Putamen | T(1510) = 2.22 | 0.027 | R SMA-L Thalamus | T(1510) = 3.11 | 0.002 |
| R S1-L Thalamus | T(1510) = 2.45 | 0.014 | R S1-R Pallidum | T(1510) = 2.62 | 0.009 |
| R S1-L Pallidum | T(1510) = 2.39 | 0.017 | L SMA-L Pallidum | T(1510) = 2.72 | 0.007 |
| R S1-R Thalamus | T(1510) = 2.31 | 0.021 | L SMA-R Thalamus | T(1510) = 2.62 | 0.009 |
| R S1-R Pallidum | T(1510) = 2.20 | 0.028 | L SPL-R Thalamus | T(1510) = 2.91 | 0.004 |
| L S1-R Putamen | T(1510) = 2.13 | 0.033 | L M1-R Pallidum | T(1510) = 2.37 | 0.018 |
| R SMA-L Caudate | T(1510) = -2.19 | 0.029 | L S1-R Pallidum | T(1510) = 2.48 | 0.013 |
| R SMA-R Caudate | T(1510) = -2.10 | 0.036 | R SMA-L Pallidum | T(1510) = 2.48 | 0.013 |
|  | | | R SMA-R Pallidum | T(1510) = 2.25 | 0.024 |
|  |  |  | R SMA-R Thalamus | T(1510) = 2.17 | 0.030 |
|  |  |  | L S1-L Pallidum | T(1510) = 2.23 | 0.026 |
|  |  |  | L SPL-L Thalamus | T(1510) = 2.56 | 0.010 |
|  |  |  | L SMA-R Pallidum | T(1510) = 2.30 | 0.021 |
|  |  |  | R M1-R Putamen | T(1510) = 1.98 | 0.048 |
|  | | | | | |
| Subcortical-Subcortical Cluster | F(_4,1507)_ = 2.93 | 0.019 |  | | |
| L Thalamus-L Putamen | T(1510) = 2.06 | 0.039 |  |  |  |
| L Thalamus-R Thalamus | T(1510) = -2.01 | 0.045 |  |  |  |
| R Caudate-L Pallidum | T(1510) = 2.64 | 0.008 |  |  |  |
| R Caudate-R Pallidum | T(1510) = 2.44 | 0.015 |  |  |  |
|  | | | | | |
| **Cur with PmR-HC** |  |  | **Cur with PmA** |  |  |
| Connection | Statistic | p-value | Connection | Statistic | p-value |
| Cortico-Cortical Connections |  |  |  | | |
| L S1-R SMA | T(1510) = 2.50 | 0.012 |  |  |  |
| R S1-R SMA | T(1510) = 1.99 | 0.047 |  |  |  |
| L M1-R SMA | T(1510) = 2.15 | 0.032 |  |  |  |
|  | | | | | |
| Cortical-Subcortical Connections |  |  | Cortical-Subcortical Cluster | F(_4,1507)_ = 3.43 | 0.009 |
| L S1-R Thalamus | T(1510) = 4.26 | 0.000 | R S1-L Pallidum | T(1510) = 3.08 | 0.000 |
| L S1-L Thalamus | T(1510) = 3.46 | 0.001 | L M1-L Pallidum | T(1510) = 2.95 | 0.003 |
| R S1-R Thalamus | T(1510) = 3.84 | 0.000 | L S1-L Pallidum | T(1510) = 2.80 | 0.005 |
| R S1-L Thalamus | T(1510) = 3.65 | 0.000 | L S1-R Pallidum | T(1510) = 2.71 | 0.007 |
| R Thalamus-L S1 | T(1510) = 4.26 | 0.000 | R M1-L Pallidum | T(1510) = 2.83 | 0.005 |
| R Thalamus-R S1 | T(1510) = 3.84 | 0.000 | L S1-L Thalamus | T(1510) = 2.33 | 0.020 |
| R Thalamus-L M1 | T(1510) = 3.36 | 0.001 | L SPL-R Thalamus | T(1510) = 2.35 | 0.019 |
| R Thalamus-R M1 | T(1510) = 3.16 | 0.002 | L SPL-L Thalamus | T(1510) = 2.28 | 0.023 |
| R Thalamus-R SPL | T(1510) = 2.38 | 0.017 | L SPL-R Pallidum | T(1510) = 2.13 | 0.033 |
| R Thalamus-L SMA | T(1510) = 2.36 | 0.018 | L SPL-R Caudate | T(1510) = 2.13 | 0.034 |
| R Thalamus-R SMA | T(1510) = 2.00 | 0.045 | L M1-R Putamen | T(1510) = 2.39 | 0.017 |
| L SPL-R Caudate | T(1510) = -2.08 | 0.038 | L M1-R Cerebellum 4/5 | T(1510) = -2.29 | 0.022 |
| L M1-R Thalamus | T(1510) = 3.36 | 0.001 | L S1-R Thalamus | T(1510) = 2.17 | 0.030 |
| L M1-L Thalamus | T(1510) = 3.11 | 0.002 | R S1-R Pallidum | T(1510) = 2.43 | 0.015 |
| R SPL-R Thalamus | T(1510) = 2.38 | 0.017 | R SPL-L Thalamus | T(1510) = 2.57 | 0.010 |
| R SPL-R Caudate | T(1510) = -2.24 | 0.025 | R SPL-R Thalamus | T(1510) = 2.31 | 0.021 |
| R SPL-L Thalamus | T(1510) = 2.09 | 0.037 | L SPL-L Pallidum | T(1510) = 1.98 | 0.048 |
|  | | | L M1-R Pallidum | T(1510) = 2.13 | 0.034 |
|  |  |  | R M1-R Putamen | T(1510) = 2.18 | 0.029 |
|  |  |  | R M1-R Pallidum | T(1510) = 2.13 | 0.034 |
|  |  |  | R M1-R Thalamus | T(1510) = 2.08 | 0.037 |
|  |  |  | R S1-L Thalamus | T(1510) = 2.02 | 0.044 |
|  |  |  | R S1-R Thalamus | T(1510) = 2.00 | 0.046 |
|  |  |  | L SMA-R Putamen | T(1510) = 2.22 | 0.026 |
|  |  |  | R SMA-L Pallidum | T(1510) = 1.99 | 0.046 |
|  | | | | | |
| Subcortical-Subcortical Connections |  |  | Subcortical-Subcortical Cluster | F(_4,1507)_ = 3.07 | 0.016 |
| R Thalamus-R Putamen | T(1510) = 2.24 | 0.025 | R Caudate-R Putamen | T(1510) = -3.65 | 0.000 |
|  | | | R Caudate-L Putamen | T(1510) = -3.28 | 0.001 |
|  |  |  | L Caudate-L Putamen | T(1510) = -2.73 | 0.006 |
|  |  |  | L Caudate-R Putamen | T(1510) = -2.65 | 0.008 |
|  |  |  | L Thalamus-L Caudate | T(1510) = -2.44 | 0.015 |
|  |  |  | R Thalamus-L Caudate | T(1510) = -2.19 | 0.029 |

| **Table C2: Between-group differences in graph measures** | | | | | | | | |
| --- | --- | --- | --- | --- | --- | --- | --- | --- |
|  | Global Efficiency | | | | Clustering Coefficient | | | |
| Contrast | Δβ | t-value | p-value | pFDR | Δβ | t-value | p-value | pFDR |
| HC-MDD | 0.007 | 1.891 | 0.059 | 0.127 | -0.007 | -1.053 | 0.292 | 0.515 |
| HC-cur | 0.008 | 1.694 | 0.090 | 0.180 | -0.008 | -1.008 | 0.314 | 0.517 |
| HC-rem | 0.006 | 1.413 | 0.158 | 0.295 | -0.005 | -0.734 | 0.463 | 0.589 |
| cur-rem | -0.002 | -0.373 | 0.709 | 0.812 | 0.003 | 0.309 | 0.379 | 0.559 |
| ***HC-curnonPmD*** | ***0.020*** | ***3.074*** | ***0.002*** | **0.014** | *-0.023* | *-2.070* | *0.039* | 0.109 |
| HC-curPmD | -0.002 | -0.260 | 0.795 | 0.856 | 0.006 | 0.618 | 0.268 | 0.515 |
| HC-curPmR | -0.004 | -0.492 | 0.622 | 0.792 | 0.007 | 0.542 | 0.294 | 0.515 |
| HC-curPmA | -0.004 | -0.375 | 0.708 | 0.812 | 0.004 | 0.208 | 0.418 | 0.580 |
| ***curPmD-curnonPmD*** | ***0.022*** | ***2.645*** | ***0.008*** | **0.037** | *-0.029* | *-2.113* | *0.035* | 0.109 |
| HC-curPmM | 0.010 | 0.610 | 0.540 | 0.784 | 0.010 | 0.340 | 0.730 | 0.840 |
| ***curPmR-curnonPmD*** | ***0.024*** | ***2.474*** | ***0.013*** | **0.046** | -0.030 | -1.841 | 0.066 | 0.168 |
| *curPmA-curnonPmD* | *0.024* | *1.964* | *0.050* | 0.117 | -0.027 | -1.284 | 0.199 | 0.429 |
| curPmM-curnonPmD | -0.010 | -0.910 | 0.360 | 0.630 | 0.030 | 1.300 | 0.190 | 0.429 |
| curPmM-curPmA | -0.010 | -0.740 | 0.461 | 0.717 | <0.001 | -0.110 | 0.911 | 0.945 |
| curPmM-curPmR | -0.010 | -0.830 | 0.407 | 0.670 | <0.001 | 0.020 | 0.980 | 0.980 |
| curPmR-curPmA | <0.001 | -0.010 | 0.990 | 0.990 | <0.001 | -0.160 | 0.870 | 0.937 |
| ***remPmR-remPmA*** | *-0.040* | *-2.300* | *0.020* | 0.051 | ***0.080*** | ***2.580*** | ***0.010*** | **0.040** |
| HC-remnonPmD | 0.003 | 0.583 | 0.560 | 0.784 | 0.001 | 0.163 | 0.435 | 0.580 |
| ***HC-remPmD*** | ***0.025*** | ***2.766*** | ***0.006*** | **0.034** | ***-0.045*** | ***-2.927*** | ***0.003*** | **0.014** |
| ***HC-remPmR*** | ***0.045*** | ***3.619*** | ***<0.001*** | **<0.001** | ***-0.084*** | ***-3.959*** | ***<0.001*** | **<0.001** |
| HC-remPmA | 0.005 | 0.352 | 0.725 | 0.812 | -0.007 | -0.319 | 0.750 | 0.840 |
| ***remPmD-remnonPmD*** | *-0.022* | *-2.351* | *0.019* | 0.051 | ***0.046*** | ***2.869*** | ***0.002*** | **0.014** |
| ***remPmR-remnonPmD*** | ***-0.042*** | ***-3.306*** | ***0.001*** | **0.009** | ***0.085*** | ***3.913*** | ***<0.001*** | **<0.001** |
| remPmA-remnonPmD | -0.002 | -0.140 | 0.889 | 0.922 | 0.008 | 0.368 | 0.356 | 0.554 |
| ***curnonPmD-remnonPmD*** | ***-0.017*** | ***-2.444*** | ***0.015*** | **0.047** | *0.024* | *2.003* | *0.023* | 0.081 |
| ***curPmD-remPmD*** | ***0.027*** | ***2.571*** | ***0.010*** | **0.040** | ***-0.051*** | ***-2.933*** | ***0.003*** | **0.014** |
| ***curPmR-remPmR*** | ***0.049*** | ***3.398*** | ***0.001*** | **0.009** | ***-0.091*** | ***-3.730*** | ***<0.001*** | **<0.001** |
| curPmA-remPmA | 0.009 | 0.518 | 0.605 | 0.792 | -0.011 | -0.383 | 0.702 | 0.840 |
| *Italics*: significant at uncorrected p<0.05*.* **Bold:** significant at FDR-corrected q<0.05. | | | | | | | | |

| Table C3: Resting-state BOLD ROI-to-ROI functional connectivity within the cerebral motor network association with symptom severity | | | | | |
| --- | --- | --- | --- | --- | --- |
| **Association with HAMD** | | | **Association with GAF** | | |
| Connection | Statistic | p-value | Connection | Statistic | p-value |
| Subcortical-Subcortical Cluster | F(2,690) = 7.45 | 0.009 | Cerebellum-Cerebellum Clusters | F(1,681) = 8.58 | 0.026 |
| R Thalamus-R Caudate | T(691) = -3.57 | 0.006 | R Cereb 4/5-L Cereb4/5 | T(681) = 2.93 | 0.023 |
| R Thalamus-L Caudate | T(691) = -3.41 | 0.006 | Cortical-Cortical Cluster | F(2,680) = 7.57 | 0.008 |
| L Thalamus-R Caudate | T(691) = -3.53 | 0.007 | R M1- R SMA | T(681) = -3.5 | 0.006 |
| L Thalamus-L Caudate | T(691) = -3.22 | 0.011 | L M1- R SMA | T(681) = -3.19 | 0.023 |
|  | | | R M1- L SMA | T(681) = -2.73 | 0.056 |
|  |  |  | R S1- R SMA | T(681) = -3.06 | 0.039 |
|  |  |  | L S1- R SMA | T(681) = -3.10 | 0.034 |

| Table C4: Association between depression and functioning severity in graph theory measures | | | | | | |
| --- | --- | --- | --- | --- | --- | --- |
|  | Global Efficiency | | | | Clustering Coefficient | |
| Contrast | β | t-value | p-value | β | t-value | p-value |
| Association with HAMD | 0.000 | -1.07 | 0.283 | 0.00 | 0.98 | 0.327 |
| Association with GAF | 0.000 | 2.45 | 0.014 | 0.000 | -1.95 | 0.05 |
| Association between depression and functioning severity of graph measures adjusted for age, sex, MR-hardware and software changes, and in-scanner movement. *Italics*: significant at uncorrected p<0.05*.* **Bold:** significant at FDR-corrected q<0.05.  *HAMD: Hamilton Depression Rating Scale, GAF: Global Assessment of Functioning* | | | | | | |
